# Applying a Method to Estimate the Breeding and Non-Breeding Population Fractions of the Globally Threatened Red-Spectacled Amazon

**DOI:** 10.3390/biology15020190

**Published:** 2026-01-20

**Authors:** José L. Tella, Jaime Martínez, Francisco V. Dénes, Viviane Zulian, Fernando Hiraldo, Nêmora P. Prestes

**Affiliations:** 1 Department of Conservation Biology and Global Change, Estación Biológica de Doñana (EBD-CSIC), 41092 Sevilla, Spain; hiraldo@ebd.csic.es; 2 Departamento de Vida Silvestre Projeto Charão (AMA), Universidade de Passo Fundo (UPF), Passo Fundo 99052-900, Brazil; martinez@upf.br (J.M.); prestes@upf.br (N.P.P.); 3 Department of Ecology, Biosciences Institute, University of São Paulo, São Paulo 05508-090, Brazil; francisco.denes@ib.usp.br; 4 Programa de Pós-Graduação em Ecologia, Universidade Federal de Santa Catarina (UFSC), Florianópolis 88040-900, Brazil; zulian.vi@gmail.com

**Keywords:** *Amazona pretrei*, *Araucaria angustifolia*, cryptic populations, extinction risk, non-breeding populations, population size, population monitoring, Psittaciformes, red-spectacled amazon

## Abstract

Population size estimates are essential for investigating numerous aspects of the ecology, evolution, and conservation of wildlife. However, the life cycles of many species include cryptic life stages that are difficult to detect or sample, such as the non-breeding fractions typical of many bird populations. Quantifying the proportions of breeders and non-breeders is crucial to better assess their conservation status and population trends. We propose a simple method applicable to species that show phenotypic differences between adults and juveniles. By quantifying the proportion of ages, the size of the whole population, and its productivity, the size of the non-breeding and breeding fractions can be inferred. We applied this approach to the red-spectacled amazon, *Amazona pretrei*, a threatened parrot endemic to the Brazilian Atlantic Forest. The entire global population aggregates in winter in a few localities to feed on the seeds of the Parana pine *Araucaria angustifolia*, a critically endangered tree whose range has declined >97% due to massive exploitation. From a global population of ~16,000 individuals in 2015 and ~20,000 individuals in 2017, our methodology allowed us to estimate a low proportion of juveniles (14%) and a large proportion of non-breeders (80%) within the adult population, with narrow confidence intervals. These proportions did not change between years, but the estimated number of breeding pairs increased, from ca. 1300 to 1700 pairs, in parallel with the global population increase. Our methodology, with its possible improvements to reduce uncertainty in estimates, can be applied to the long-term monitoring of this and many other species, to better understand their conservation challenges and inform effective management strategies.

## Simple Summary

Information on individuals from all stages of life is crucial for advancing knowledge of the ecology, evolution, and conservation of species. However, the life cycles of many species include cryptic stages that are often overlooked in population studies, such as the non-breeding fractions in bird populations. Here, we present a simple method applicable to species that show phenotypic differences between adults and juveniles. By quantifying age proportions, total population size, and productivity, we can infer the sizes of the non-breeding and breeding fractions. We applied this approach to a threatened parrot species whose global population concentrates in a small region of Southern Brazil after breeding. Our results indicate a low proportion of juveniles (14%) and a high proportion of non-breeders (80%) within the adult population, from a global population ranging between 16,000 and 20,000 individuals. This method can be applied to long-term monitoring of this and many other species, improving our understanding of their conservation challenges and informing effective management strategies.

## 1. Introduction

Determining the size of animal populations is crucial for numerous studies on their ecology, evolution, or conservation. In fact, estimates of population sizes inform a range of decision-making processes such as the IUCN Red Listing, which in turn underpins many conservation prioritization processes [1]. Estimating population sizes is a challenge, and in the case of birds, most studies focus on breeding populations or whole population sizes. However, little attention has been given to the non-breeding fractions of wild populations, despite their increasingly recognized importance in population size and in improving our understanding of population dynamics and conservation challenges [2]. For example, breeding and non-breeding fractions of a single population may differ in the threats they face [3,4], which may be reflected in different population dynamics [5,6]. On the other hand, IUCN uses, among other criteria, estimates of the number of mature individuals as a proxy of effective population sizes (N_e_) for identifying globally threatened species to be included in the Red List [7,8]. This information most often relies on very crude estimates and inter-species extrapolations, given the difficulties for obtaining the size of non-breeding populations, to the extent that these fractions have been termed “cryptic” or “ghost” populations [9,10]. Inaccuracies in estimating the breeding to non-breeding population rates [9] may lead to misleading conservation status and actions [4]. Therefore, a great effort is needed to advance our knowledge of population structures, not only through increasing monitoring efforts, but also by relying on newly proposed tools, such as population modeling [11,12] and non-invasive genetic tagging [13].

In the case of species with phenotypic differences between juveniles and adults, simple population surveys conducted after the breeding season, combined with information on population size and breeding success, may offer reliable information on the proportions of adults/juveniles and breeding/non-breeding adults, as exemplified for a restricted-ranged macaw species [4]. Populations of parrots (order Psittaciformes) should be expected to include a large fraction of non-breeders, since they usually are long-lived species with deferred sexual maturity [14]. In fact, in three parrot species studied, it was found that the fraction of the non-breeding population was greater than the breeding fraction [4,15,16]. However, much more information is needed before patterns on breeding to non-breeding population ratios can be generalized. This is especially necessary in the case of parrots, one of the most diverse groups of birds (with around 400 species), yet also among the most threatened with extinction [17,18], and for which information on their abundance and population sizes remains scarce [19].

**Figure 1:**
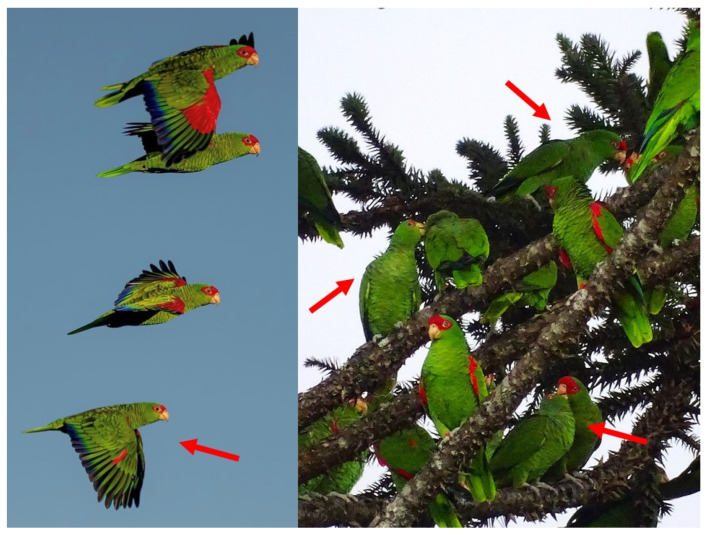
Juvenile red-spectacled amazons (indicated with red arrows) show much smaller plumage red patches in the head and wings than adults, both in flight and when they are perched. Photographs: Archive Projeto Charão.

Here, we essayed a simple method applicable to species that show phenotypic differences between adults and juveniles, to estimate age proportions and breeding to non-breeding population fractions in the red-spectacled amazon (*Amazona pretrei*). This is a globally threatened parrot, endemic to the Atlantic Forest of southern Brazil. The species is listed by IUCN as Vulnerable worldwide [20] and in Brazil [21], due to a large population decline and range contraction. The red-spectacled amazon is distributed over only about 24,600 km^2^, and its distribution contracts even further in winter [20,22], when the species aggregates in a few localities to feed almost exclusively on the seeds of the Parana pine *Araucaria angustifolia* [23,24]. The Parana pine is listed by IUCN as critically endangered, given that its range declined by more than 97% in the last century, due to massive exploitation for timber, generating a mosaic of forest patches [25]. These parrots are the main legitimate dispersers of the seeds of the Parana pine, so the conservation of the two species is closely intertwined [26]. However, seed predation by non-native mammals reduces the potential regeneration of Parana pine forests as well as food supply for their native consumers [27]. Although the population of red-spectacled amazon has remained relatively stable in recent years [20,22], less than 1% of its year-round distribution is covered by protected areas [28]. Moreover, niche modeling predicts drastic decreases for breeding (63%) and wintering (91%) ranges by 2060 [28]. Given the species’ precarious conservation status, it is important not only to estimate total population size but also to quantify its breeding and non-breeding fractions in order to better understand threats and identify appropriate management actions [4]. We demonstrate how these demographic components can be derived for the global population of the species by leveraging plumage-based age distinctions between adults and juveniles (Figure 1) in combination with long-term monitoring of population size and breeding parameters.

## 2. Materials and Methods

### 2.1. Study Species and Study Area

The red-spectacled amazon is a medium-sized parrot, with adults showing large red patches in the forecrown to lores and around the eyes, as well as on the bend of the wing to carpal edge. Juveniles have a distinctive plumage, with much smaller red patches restricted to the forehead and the bend of the wing [29]. These age-related plumage differences are readily discerned in the field, whether birds are perched or in flight (Figure 1).

**Figure 2:**
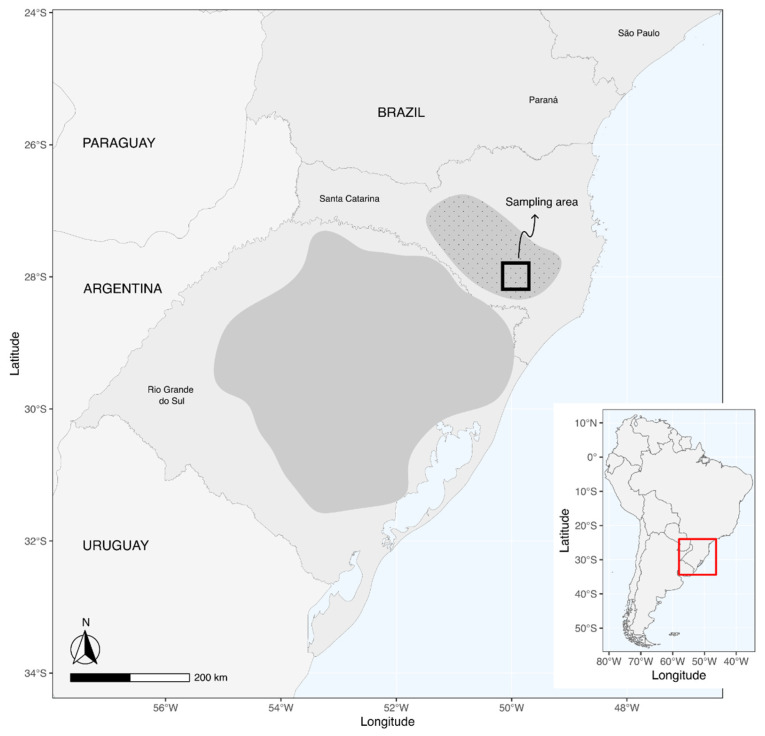
Global breeding (solid gray polygon) and non-breeding (wintering) range (dotted gray polygon) of the red-spectacled amazon (*Amazona pretrei*), according to the IUCN Red List [20]. The black square shows the study area, mostly covering the municipalities of Painel, Bocaina do Sul, and Urupema, where the world population concentrates during May and June in recent decades.

Pairs are monogamous and breed dispersedly in cavities of a variety of tree species [30]. A few months after chicks fledge, all individuals concentrate in forests of Parana pine, where they spend the winter in large flocks, feeding mostly on the large seeds of this critically endangered tree species [24]. The winter distribution of red-spectacled amazon has contracted recently [28], and for the past two decades, nearly the entire population concentrates during May-June in the small municipalities of Painel, Bocaina do Sul, and Urupema, in the Serra Catarinense region of Santa Catarina state, Brazil ([23,31], N.P. Prestes and J. Martinez unpubl. data) (black square in Figure 2). During this period, the parrots gather in a large communal roost, facilitating census and allowing annual assessments of the global population size since 1995 ([22,23,31], J. Martinez and N.P. Prestes, unpubl. data).

### 2.2. Field Surveys

Field work specific to this study was conducted in May 2015 and 2017 in the Serra Catarinense (Figure 2). Censuses of amazons gathering at the large communal roost were systematically carried out every year since 1995 by NPP and JM, with the help of experienced volunteers, and results were published elsewhere [22,23,31]. This is a clear example where entire parrot populations can be counted when they congregate in a few communal roosts that are reused year after year [32]. Briefly, the observers positioned at four fixed points, sited at North, South, East, and West advantage points surrounding the roosting site, and counted all parrots arriving at the roost with the help of binoculars. Observers recorded the arrival time, flock size, incoming flight direction (N, S, W, E), and their destination within the roosting site. Roost surveys began one hour before sunset and extended for thirty minutes afterward. At the end, the observers compared the information regarding the arrival times and flight directions of the flocks to avoid double-counting instances. These censuses were repeated for five consecutive days (between 1 and 5 May), and the maximum value obtained was taken as the global population size.

Poor light conditions (i.e., twilight) during roost censuses hindered differentiation of age-related plumage patterns. We thus relied on roadside car surveys, which maximize the probability of encountering flocks of parrots that are scarce and scattered in the landscape [33]. JM first designed, and traveled with the rest of the authors on 24 May, an itinerary of 105 km of unpaved roads that covered well the main foraging areas of the species in the Serra Catarinense. Thereafter, this itinerary was surveyed by a team (FD, VZ, FH, and JLT) by driving a car at low speed (20–40 km/h) between 25 and 28 May. These roadside surveys were conducted within a radius of approximately 50 km around the communal roost. The location of the communal roost and of these surveys covering the foraging areas is not detailed in Figure 2, as this is sensitive information whose disclosure could negatively affect the conservation of the species. Roadside parrot surveys allow for sampling large areas during a short period of time, thus reducing the probability of observing the same individuals twice [33]. The probability of resampling the same individuals also seems very low, considering that we sampled only 4–7% of the global population to obtain the age proportion (see Section 3).

We stopped the car each time we saw or heard amazons to locate them and identify the age of individuals observed. In these cases, one or two observers spotted perching birds with binoculars and a 20–50× telescope, while another observer attempted to take photographs of flying flocks with a 300–400 mm camera lens, and another recorded the data. We recorded the age of observed birds in situ, while we further identified the ages in photographs on computer screens. Following this successful experience, in 2017, we only took photos of different parrot flocks to estimate the age ratio on the same dates.

### 2.3. Data Analysis

We estimated productivity—defined as the average number of fledglings per breeding attempt, including failures—by monitoring 49 breeding events over 24 years (period 1994–2017) ([30,34], N.P. Prestes and J. Martínez, unpubh. Data), yielding an average of 1.63 (SD = 1.42, range = 0–4, 95% CI: 1.23–2.02) fledglings per nest. Attending to this value, we tested the statistical significance of the proportion of adults observed during the field surveys against the expected value of 1.23 adults per juvenile bird (i.e., 2 adult parents per 1.63 fledglings). We extrapolated the obtained age proportions to the whole population count to estimate the total number of juveniles and adults. We estimated the number (and proportion) of adult breeders as 2 parents × (number of juveniles/average productivity), and the number (and proportion) of breeding pairs as the number of estimated breeding adults divided by 2. We calculated the number (and proportion) of non-breeding adults as the total number of adults − number of breeding adults. We assessed the uncertainty of estimates obtained for age proportions, breeding and non-breeding fractions, and number of breeding pairs as their 95% confidence intervals (95% CI). We performed separate calculations for 2015 and 2017. We used Yates’ corrected Chi-square tests for testing differences in proportions between observed and expected ages and between years.

## 3. Results

In 2015, we estimated the global population of red-spectacled amazons at 15,685 individuals, of which we were able to identify the age of 1097 individuals (7% of the total) belonging to 60 different flocks, and composed mostly by adults (N = 948). The obtained proportion of adults in the population (86.42%) showed a low margin of uncertainty (95%CI: 84.39–88.44%) and differed significantly from the expected proportion (ꭕ^2^ = 149.36, df = 1, *p* < 0.0001), considering the expected value of 1.23 adults per juvenile bird (i.e., 2 adult parents per 1.63 fledglings). The proportion of juveniles was 13.58%, also showing a low margin of uncertainty (95%CI = 11.56–15.61%).

Attending to proportions obtained above, 2130 of 15,685 individuals would be juveniles (95%CI: 1813–2448 individuals), and 13,555 of them would be adults (95%CI: 13,460–13,872 individuals). Considering the average productivity of 1.63 juveniles per breeding pair, and the estimated number of 2130 juveniles, there would be 1307 breeding pairs (95% CI: 1112–1502 breeding pairs), equaling to 2614 breeding adults (95%CI: 2224–3004 breeding adults) in the whole population. Therefore, the breeding fraction of the adult population would be 19.28% (95%CI: 16.03–22.69%), and the non-breeding fraction of adults would reach 80.71% (95%CI: 77.30–83.97%). These proportions remained similar when obtained by applying the extreme values of the 95%CI of the productivity recorded for the species (Table 1).

In 2017, the estimated global population reached 20,128 individuals. We were able to determine the age of 918 individuals (4.56% of the total), again finding a proportion of adults (85.51%, 95%CI: 83.24–87.79%) and juveniles (14.49%, 95%CI = 12.21–16.76%) that differed significantly from those expected given the average productivity of the species (ꭕ^2^ = 119.47, df = 1, *p* < 0.0001). These age proportions did not differ from those recorded in 2015 (ꭕ^2^ = 0.269, df = 1, *p* = 0.604). Consequently, the non-breeding and breeding adult population fractions remained almost identical (Table 1), with the number of breeding pairs (1789, 95%CI: 1508–2070) increasing in parallel with the overall population size. As in 2015, the proportions of breeding and non-breeding adults remained similar when obtained by applying the extreme values of the 95%CI of the productivity recorded for the species (Table 1).

## 4. Discussion

Few attempts have been made to estimate the non-breeding fraction of bird populations, despite its key importance in ecology and conservation [2,12]. To our knowledge, breeding to non-breeding ratios were previously obtained for only three species of Psittaciformes. For those species, it was possible to quantify both the number of breeding pairs and the total population [4,15,16], as they nest colonially on cliffs and aggregate at predictable communal roosts, which facilitates population censuses [32]. Here, we present a simple method, applicable to species with phenotypic differences between adults and juveniles, which allowed us to infer the size of the non-breeding fraction by quantifying the proportion of ages, the size of the whole population, and its productivity. This method is especially valuable for species for which censusing breeding populations is difficult or logistically unfeasible, requiring the use of indirect methods [32,35], such as the red-spectacled amazon, whose pairs nest in a scattered manner and at low densities across large forest areas [23,30,34].

Our results show a low percentage of juveniles in the population and a large proportion of non-breeding adults. These findings are consistent with the life-history theory, since most parrots are long-lived species, with a slow breeding strategy, deferred sexual maturity, long life spans, and a relatively long period of senescence [14]. Therefore, an important fraction of the adult population is expected to be composed of non-breeding individuals, which may include immature birds with adult plumage, unpaired mature birds, and even senescent ones (i.e., infertile old individuals). The non-breeding fraction of adults obtained (ca. 80%) is similar to that obtained for the red-fronted macaw *Ara rubrogenys* [4] and Lear’s macaw *Anodorhynchus leari* [15], but much higher than that obtained for the burrowing parrot *Cyanoliseus patagonus* (ca. 40–50%) [16]. Although information from wild populations is lacking, these results agree with data from captive birds, which indicate a higher age of first reproduction and longer senescence periods in macaws and amazons than in other smaller-sized, shorter-lived parrot species [14]. Future studies focused on several species covering a greater variability of life histories [14] are needed to test the hypothesis that species with longer life expectancy exhibit a higher proportion of non-breeding adults.

Besides the life history traits of each species, ecological conditions may also increase the non-breeding fraction of populations. Many parrot species are obligate secondary cavity nesters, and habitat loss may reduce breeding densities through a reduction in tree cavities and an increase in interspecific competition for nest sites [36]. The drastic reduction in forests inhabited by red-spectacled amazons in recent decades [20,25,28] may have limited the availability of nest cavities in the remaining old-growth forests and thus increased the fraction of non-breeding adults. However, this fraction could have been unchanged given that the global population of the species also apparently declined in parallel to habitat loss [20]. The addition of artificial nest boxes may serve as an experiment for testing whether nest sites constitute a limiting resource for breeding populations [36,37]. The installation of nest-boxes in six regions within the current breeding distribution of the red-spectacled amazon has shown a low percentage of occupation by this species (3.6%) [38], suggesting that breeding numbers are not strongly limited by nest-site availability. Similarly, the apparent increase in the number of breeding pairs between 2015 and 2017, in parallel with a global population increase that has continued in subsequent years ([22], N.P. Prestes and J. Martínez, unpubh. Data), does not suggest a strong limitation of nesting sites at present.

The reliability of population structure estimates may be strongly influenced by the strength of surveys related to the overall population size. In our case, we were able to age approximately 1000 individuals each year (i.e., 4–7% of the global population), with a low probability of pseudoreplication given our sampling design, resulting in reliable estimates of the juvenile-to-adult ratio with narrow confidence intervals. The high consistency of the age proportions obtained over two years with different population sizes generates additional confidence in the methodology used. On the other hand, the proportion of breeding versus non-breeding adults is markedly influenced by estimates of productivity (number of fledglings per breeding attempt). The relatively low number of breeding attempts we monitored may increase the uncertainty in our estimate. Ideally, robust information on breeding parameters should be obtained from the breeding season preceding the population survey; however, achieving large sample sizes within a single year is challenging for threatened species such as our study model. Nonetheless, our productivity estimates were derived from 24 years of breeding monitoring, likely capturing interannual variations, and thus may approximate the true parameter value reasonably well. However, our methodology does not account for juvenile mortality between fledgling and survey time, which may be higher than adult mortality during the same period [6]. By underestimating the proportion of juveniles, this limitation could lead to negative bias in the estimates of breeding adult fractions. Finally, roost monitoring programs can also be refined to account for the uncertainty in counts and population size estimates [39]. Improving the quality of reproductive and population parameters would further increase the robustness of age structure and breeding-to-non-breeding adult fraction estimates. Even recognizing the methodological limitations inherent in each population studied, the systematic replication of surveys over multiple years can provide reliable insights into long-term changes in the population structure of species.

## 5. Conclusions

Our proposed methodology can be applied to any species that exhibits age-related plumage dimorphism and for which estimates of the entire population size and breeding success are available. In this study, this method allowed us to obtain reliable estimates for the global population of a parrot species, facilitated by its restricted geographic range and the concentration of individuals in a few well-defined communal roosts. However, this approach can also be applied to local populations of other species, provided that they function as closed populations and that age classes are not spatially structured (e.g., juveniles and/or non-breeders concentrate out of the surveyed breeding population).

## Figures and Tables

**Table 1:** Estimated global population size, number of individuals sampled for age determination, percentage of juveniles and adults recorded among them, and the proportion of non-breeding and breeding adults, and the number of breeding pairs estimated in 2015 and 2017. Numbers in parentheses represent the 95% confidence intervals (CI). We obtained the estimates of non-breeding and breeding fractions by applying the average, lower, and upper 95%CI values of productivity (number of fledglings/nest) recorded for the species.

Productivity	Average = 1.63	Lower 95% = 1.23	Upper 95% = 2.02
2015			
Global population	15,685		
Population sampled	1097		
% Juveniles	13.58 (11.56–15.61)		
% Adults	86.42 (84.39–88.44)		
% Non-breeding adults	80.71 (77.30–83.97)	74.41 (69.93–78.75)	84.45 (80.19–87.05)
% Breeding adults	19.28 (16.03–22.69)	25.58 (21.25–30.07)	15.55 (12.95–19.81)
Number of breeding pairs	1307 (1112–1502)	1732 (1474–1990)	1054 (898–1212)
2017			
Global population	20,128		
Population sampled	918		
% Juveniles	14.49 (12.21–16.76)		
% Adults	85.51 (83.24–87.79)		
% Non-breeding adults	79.21 (74.11–82.93)	72.45 (65.70–77.40)	82.23 (79.11–86.24
% Breeding adults	20.79 (17.07–25.89)	27.55 (22.60–34.30)	16.77 (13.76–22.89)
Number of breeding pairs	1789 (1508–2070)	2371 (1997–2742)	1443 (1216–1670)

## Data Availability

All data used for analyses are provided in the body of the article.
